# High-pressure polymorphism in pyridine

**DOI:** 10.1107/S2052252519015616

**Published:** 2020-01-01

**Authors:** Nico Giordano, Christine M. Beavers, Branton J. Campbell, Václav Eigner, Eugene Gregoryanz, Willliam G. Marshall, Miriam Peña-Álvarez, Simon J. Teat, Cara E. Vennari, Simon Parsons

**Affiliations:** a Centre for Science at Extreme Conditions and EastChem School of Chemistry, University of Edinburgh, Edinburgh EH9 3FJ, UK; b Advanced Light Source, Lawrence Berkeley National Laboratory, Berkley, CA 94720, USA; c Department of Earth and Planetary Sciences, University of California, Santa Cruz, CA 95064, USA; d Diamond Light Source, STFC Rutherford Appleton Laboratory, Harwell Science and Innovation Campus, Didcot OX11 0QX, UK; e Department of Physics and Astronomy, Brigham Young University, Provo, UT 84602, USA; f Institute of Physics of the AS CR, v.v.i., Cukrovarnicka 10, 162 00 Prague 6, Czech Republic; g School of Physics and Astronomy and the Centre for Science at Extreme Conditions, University of Edinburgh, Edinburgh EH9 3FD, UK; h ISIS Neutron and Muon Source, Science and Technology Facilities Council, Rutherford Appleton Laboratory, Harwell, Didcot OX11 0QX, UK

**Keywords:** polymorphism, pressure, *in situ* crystallization, phase transitions

## Abstract

Single crystals of the high-pressure phases II and III of pyridine have been obtained by *in situ* crystallization at 1.09 and 1.69 GPa, revealing the crystal structure of phase III for the first time using X-ray diffraction. Phase II crystallizes in *P*2_1_2_1_2_1_ with *Z*′ = 1 and phase III in *P*4_1_2_1_2 with *Z*′ = ½. Neutron powder diffraction experiments using pyridine-d_5_ establish approximate equations of state of both phases. The space group and unit-cell dimensions of phase III are similar to the structures of other simple compounds with *C*_2v_ molecular symmetry, and the phase becomes stable at high pressure because it is topologically close-packed, resulting in a lower molar volume than the topologically body-centred cubic phase II. Phases II and III have been observed previously by Raman spectroscopy, but have been mis-identified or inconsistently named. Raman spectra collected on the same samples as used in the X-ray experiments establish the vibrational characteristics of both phases unambiguously. The pyridine molecules interact in both phases through CH⋯π and CH⋯N interactions. The nature of individual contacts is preserved through the phase transition between phases III and II, which occurs on decompression. A combination of rigid-body symmetry mode analysis and density functional theory calculations enables the soft vibrational lattice mode which governs the transformation to be identified.

## Abstract 2

Pyridine phase III, which forms at 1.69 GPa, is tetragonal and related to the previously characterized orthorhombic form II by a displacive phase transition. Phase III is similar to a high-pressure form of benzene. The soft mode governing the III→II transition has been identified using a combination of symmetry mode analysis and periodic density functional theory. A combination of single-crystal X-ray diffraction and Raman spectroscopy is used to resolve ambiguities in previous work on pyridine.

## 1. Introduction   

Pyridine (C_5_H_5_N) is one of the simplest organic compounds, but its crystal structure at ambient pressure (phase I, *Pna*2_1_), determined by Mootz & Wussow (1981 ▸), is unusually complicated, with four molecules in the asymmetric unit (*Z*′ = 4). A further unusual feature is that it exhibits isotopic polymorphism (Merz & Kupka, 2015 ▸; Crawford *et al.*, 2009 ▸; Castellucci *et al.*, 1969 ▸). Phase I is the thermodynamically stable form of isotopically normal pyridine (pyridine-h_5_) between the melting point (231 K) and 5 K, and it can be cooled or heated within this temperature range without undergoing any phase transitions. The same phase is formed by pyridine-d_5_ on cooling the liquid below the melting point, and the phase can also be cooled to 5 K. However, on warming from 5 K the structure transforms to the simpler phase II (*P*2_1_2_1_2_1_, *Z*′ = 1) at 170 K. This phase can also be cooled to 5 K, but on warming above 215 K it transforms back to phase I. These observations, combined with calorimetry data, indicate that the thermodynamically stable form of pyridine-d_5_ below 215 K is phase II. When phase I persists below this temperature it is metastable, the result of the sluggishness of the I→II transition.

Phase II has never been observed for pyridine-h_5_ at ambient pressure, but because it has a smaller molar volume than phase I, it can be formed at high pressure (Crawford *et al.*, 2009 ▸). We have shown that crystal growth of pyridine-h_5_ yields phase I at 0.80 and 1.03 GPa, but phase II at 1.09 GPa (Dawson, 2003 ▸). In these experiments, single crystals of pyridine were grown by first compressing the liquid into a polycrystalline mass at the desired pressure using a diamond anvil cell. The sample was then heated to melt-back the sample to a single crystallite which was then allowed to grow as the cell cooled back to room temperature. These conditions are, at least approximately, isochoric, *i.e.* the pressure is constant during crystal growth.

By contrast, phase I was obtained at 1.23 GPa by high pressure isothermal crystal growth (Podsiadło *et al.*, 2010 ▸). In these experiments, a stable crystal–liquid equilibrium was established at 1.00 GPa and 295 K. The crystal was then allowed to grow by carefully increasing the pressure but holding the temperature constant at 295 K. The sample was found to undergo a phase transition on increasing the pressure to 2.00 GPa, which destroyed the integrity of the single crystal.

The apparent difference between our and Podsiadło and coworker’s pressure conditions is quite small in the context of the precision of the pressure measurements, typically 0.05 GPa, but the difference in the phases obtained is presumably a reflection of the isochoric versus isothermal crystal growth conditions. The crystal formed at 1.00 GPa in the isothermal experiment would have been phase I. Its persistence to 1.23 GPa is likely to be the result of the sluggishness of the phase I→II transition, which is referred to by Podsiadło *et al.*, and has also been seen in the low-temperature behaviour of pyridine-d_5_ where phase I can be supercooled below the I→II transition temperature (Crawford *et al.*, 2009 ▸).

The discoveries of isotopic polymorphism of pyridine at ambient pressure and formation of high-pressure phases were first made using vibrational spectroscopy (Castellucci *et al.*, 1969 ▸), and the response of pyridine to high pressure has been the subject of a number of more recent infra-red and Raman studies. However, the overall picture that has emerged from these studies is a somewhat confusing one partly because of inconsistent phase numbering.

In their original high-pressure Raman study, Heyns & Venter (1985 ▸) obtained a phase which they referred to as ‘phase I’ for both the h_5_ and d_5_ isotopologues at *ca* 1 GPa. On increasing the pressure they observed discontinuities in the trend of band frequencies versus pressure and the appearance of one new band in pyridine-h_5_ and two in pyridine-d_5_ which were taken to indicate that this phase transforms to a ‘phase II’ at 2 GPa.

Heyns & Venter argued that the spectra they obtained for their ‘phase I’ were too simple to be compatible with the *Z*′ = 4, *Pna*2_1_ crystal structure of pyridine which had been determined by Mootz & Wussow (1981 ▸) a few years previously. They did not assign a number to Mootz’s *Z*′ = 4 phase, and they did not claim that any of their spectra are consistent with it. In short, Heyns & Venter’s paper mentions *three* crystalline phases: a low-temperature/ambient-pressure form, another forming on crystallization at 1 GPa, which transforms to a third phase just below 2 GPa. They also mention a glassy form which is characterized by broader Raman peaks. It seems likely that Heyns & Venter’s ‘phase I’ was what is referred to as ‘phase II’ in the crystallographic work described above, meaning that their ‘phase II’ is a higher pressure form for which the crystal structure has yet to be determined.

In a later study, Fanetti *et al.* (2011 ▸) describe an investigation of pyridine up to 25 GPa, showing that above 18 GPa the compound begins to undergo a rearrangement of the primary covalent bonds in which the carbon atoms become *sp* ^3^ hybridized. The same behaviour was noted by Zhuravlev *et al.* (2010 ▸) above 22 GPa. At lower pressures, the phase Fanetti *et al.* reproducibly obtained between 1.2 and 1.3 GPa, which they label ‘phase II’, could be compressed up to 18 GPa (Fanetti *et al.*, 2011 ▸). Careful peak deconvolution enabled frequencies and intensities to be extracted for 20 out of 21 external modes. The smooth trends in the frequencies of these modes with pressure do not support the phase transitions that had been proposed at 8, 11 and 16 GPa by Zhuravlev *et al.* (2010 ▸).

Decompression to around 1 GPa (Fanetti *et al.*, 2011 ▸) found that ‘phase II’ transformed to ‘phase I’. However, although they define phases I and II to be the same as in the crystallographic work, they also claim that the phases they studied had Raman spectra which closely resemble those of Heyns & Venter (1985 ▸). Therefore it seems the phases they were in fact studying must have been the two high-pressure forms reported by Heyns & Venter, *i.e.* the crystallographers’ phase II and the structurally uncharacterized form.

We now describe the crystal structure of a second high-pressure form of pyridine, which we, and hopefully everyone else, will refer to as phase III. We go on to show that this phase was the one obtained along with phase II in the high-pressure Raman studies summarized above.

## 2. Experimental   

### 2.1. Pyridine-h_5_-III: single-crystal X-ray diffraction at 1.69 GPa   

Pyridine-h_5_ (≥99%, Sigma–Aldrich, used as received) was loaded into a Merrill–Bassett diamond anvil cell (Merrill & Bassett, 1974 ▸) along with a ruby sphere. The cell contained 0.6 mm Boehler–Almax cut diamonds (Moggach *et al.*, 2008 ▸) and a pre-indented rhenium gasket with a laser-drilled hole of diameter 0.35 mm and height 0.10 mm. Pressures were determined by the ruby fluorescence method (Forman *et al.*, 1972 ▸).

**Figure 1:**
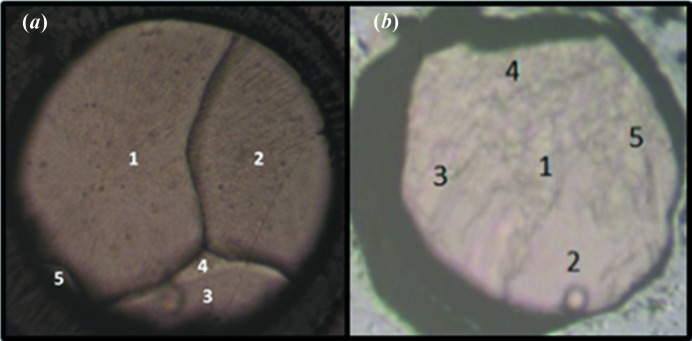
Images of the *in situ* single crystals: (*a*) phase III at 1.69 GPa, and (*b*) phase II at 1.09 GPa. The diameter of the gasket hole is 350 µm in (*a*) and 260 µm in (*b*).

Phase III was obtained by isochoric *in situ* crystallization. The DAC was pressurized to 1.55 GPa, the sample forming a polycrystalline solid. The cell was then heated using a hot plate to *ca* 400 K and slowly cooled to room temperature. This process was repeated until only large single crystals filled the sample chamber [Fig. 1 ▸(*a*)]. The crystal used for data collection, marked domain 1 in Fig. 1 ▸(*a*), accounted for about half of the sample volume. The pressure was allowed to equilibrate to 1.69 GPa prior to data collection.

Pyridine III can also be grown from methanol. The solution used for crystallization consisted of a 1:1 molar ratio of pyridine (6 ml) and methanol (3 ml). A drop of this solution was placed in a diamond anvil cell, and crystals were found to form on standing at room temperature and 1.98 GPa over the course of two days. The crystals were melted back to one seed at 423 K and the temperature was then reduced to 403 K at a rate of 2 K min^−1^ to initiate crystal growth. The temperature was then decreased to room temperature at a rate of 0.5 K min^−1^. The heating and cooling cycles were accomplished using a Cambridge Reactor Design Polar Bear heater that had been adapted to accommodate a diamond anvil cell.

### 2.2. Pyridine-h_5_-II: single-crystal X-ray diffraction at 1.09 GPa   

Pyridine-h_5_ was loaded into a Merrill–Bassett diamond anvil cell as described in Section 2.1. The cell design was similar, except that the gasket hole (diameter 0.26 mm) was formed by spark-erosion. The cavity height was 0.10 mm. The sample solidified spontaneously into large single-crystal domains on application of 1.09 GPa pressure at 293 K. X-ray diffraction data were collected on domain 2 shown in Fig. 1 ▸(*b*).

### 2.3. Data collection, processing and structure refinement   

High-pressure single-crystal X-ray diffraction data were collected at 293 K on Beamline 12.2.2 at the Advanced Light Source (Kunz *et al.*, 2005 ▸; McCormick *et al.*, 2017 ▸) on a custom-built diffractometer with Si (111) monochromated synchrotron radiation, wavelength 0.4959 Å (*E* = 25 keV), and a Perkin Elmer amorphous Si detector. Shutterless φ-scans were performed at narrow step widths of 0.25° and wide step widths of 1° in order to minimize overloads and optimize the number of reflections collected across the dynamic range of the detector. The incident beam spot size was 30 µm. Beamline calibrations were performed using a NIST single-crystal ruby standard prior to high-pressure experiments.

Data were processed using the Bruker Apex suite of programs. Dynamic masks, generated by *ECLIPSE* (Dawson *et al.*, 2004 ▸) were used to mask shaded areas of the detector and peaks were integrated to 0.8 Å using *SAINT* (Bruker, 2012 ▸). Systematic errors, including scaling, cell and sample absorption, and gasket shading were treated using the multi-scan procedure *SADABS* (Sheldrick, 2015*c* ▸). Structures were solved using dual space methods [*SHELXT*, (Sheldrick, 2015*a* ▸)] and refined by full-matrix least-squares on *F* ^2^ [*SHELXL*, (Sheldrick, 2015 ▸ *b*)] using the *OLEX2* graphical user interface (Dolomanov *et al.*, 2009 ▸). The C and N atoms were modelled with anisotropic displacement parameters. H atoms were located in Fourier difference maps, enabling the N atoms to be identified unambiguously and constrained to geometrically reasonable positions during subsequent refinement. The Flack (1983 ▸) parameter could not be reliably determined due to low completeness of the high-pressure diffraction experiments and the very small dispersion effects at the X-ray energy used for data collection. Molecular geometries were checked using *MOGUL* (Bruno *et al.*, 2004 ▸) and structures were visualized using *Mercury* (Macrae *et al.*, 2008 ▸), *DIAMOND* (Putz & Brandenburg, 1999 ▸) and *XP* (Sheldrick, 2001 ▸); application of geometric restraints was found to be unnecessary. Geometric calculations were carried out using *PLATON* (Spek, 2009 ▸). Crystal and refinement data are listed in Table 1 ▸. Crystallographic information files (CIFs) are available in the supporting information.

### 2.4. Raman spectroscopy   

High-pressure Raman spectra were collected on the same samples as used for the single-crystal data collections of pyridine-h_5_ phases II and III using a Horiba LabRAM HR Evolution Raman Spectrometer equipped with a CCD detector. Raman spectra were measured using a 633 nm excitation laser, with 1200 lines mm^−1^ grating and a spectrometer focal length of 800 mm. Spectra were collected between 50 and 3400 cm^−1^, with a resolution of 1 cm^−1^. Raman spectra were collected from a *ca* 2 µm spot size on static pressure samples at 293 K. Spectra plotted over the complete range collected are shown in Figs. S1–S4 of the supporting information. Spectra extracted from the literature were analysed using getData Graph Digitizer (v2.26).

### 2.5. Decompression of pyridine III studied by Raman spectroscopy   

A single crystal of pyridine III was grown *in situ* at 2.50 GPa from a methanol solution and its Raman spectra were collected at room temperature as the sample was decompressed to 2.44, 2.28, 1.97, 1.69 and 1.55 GPa. Pressure-dependent Raman spectra were acquired between 0 and 500 cm^−1^ using a custom-built micro-focused Raman system, using a 514 nm Argon laser as the excitation line. Raman scattering radiation was collected in back-scattering configuration. The device was equipped with a 20× Mitutoyo long-working-distance objective, three optigrate notch filters and focused onto the slit of a Princeton monochromator with a grating of 1800 grooves mm^−1^ and a CCD detector (1340 × 400 pixels). Spectra were measured with a spectral resolution of about 1–2 cm^−1^ and calibrated with a neon emission lamp.

### 2.6. Equation of state determinations by neutron powder diffraction   

A sample of pyridine-d_5_ was loaded into a null-scattering TiZr capsule together with a 1:1 by volume mixture of CaF_2_ and powdered silica. Pressure was applied at ambient temperature using a type V3b Paris–Edinburgh (PE) press (Besson & Nelmes, 1995 ▸; Besson *et al.*, 1992 ▸) in which the ram pressure was monitored and adjusted by means of a computer-controlled hydraulic system. The experiment was carried out on the PEARL instrument at the ISIS neutron spallation facility (Bull *et al.*, 2016 ▸). Pyridine is prone to texture effects, and the silica powder was included with the sample with the aim of minimizing these. A phase-pure sample of pyridine II was obtained by forming an amorphous solid at 2.7 GPa and then reducing the pressure to 0.942 (9) GPa. Further neutron powder diffraction data sets were collected at pressures of 1.09 (2), 1.22 (3), 1.59 (3), 1.83 (6) and 2.52 (5) GPa. The pattern was much weaker for the last two points, possibly because of the onset of partial amorphization. The load was reduced and further patterns collected at 1.38 (9) and 1.03 (2) GPa, the original intensity of the pattern being re-established at the second of these points. Reduction of the pressure to 0.62 (6) GPa produced a mixture of phases I and II, becoming pure phase I at 0.45 (4) GPa. The contributions of the pyridine phases to the patterns obtained were modelled with the Pawley method, while those of CaF_2_ and the WC and Ni components of the anvils were modelled with the Rietveld method [*TOPAS Academic*, (Coelho, 2018 ▸)]. The refinements yielded unit-cell volumes of CaF_2_, which were used to determine the pressures quoted above by applying a third-order Birch–Murnaghan equation of state with values for the bulk modulus (*K*_0_) and its pressure derivative (*K*′) of 81.00 GPa and 5.220, respectively (Angel, 1993 ▸).

A sample consisting of pyridine-*d*_5_ (CDN Isotopes), a small quantity of ground silica wool and a pellet of lead as a pressure marker was loaded into a TiZr capsule and pressure was applied as described above on the POLARIS instrument at ISIS. A pattern of phase III was observed at 1.599 (19) GPa. Patterns were then measured at 1.914 (18), 2.202 (14), 2.436 (18) and 2.670 (16) GPa. The sample was decompressed, with further patterns measured at 1.340 (15) and 1.229 (15) GPa. The pattern collected at 1.159 (20) GPa was a mixture of phases II and III. Data processing was as described above, the *K*_0_ and *K*′ for Pb were taken to be 41.966 GPa and 5.7167. These parameters were derived by Fortes *et al.* (2007 ▸, 2012 ▸) by refitting data obtained in three earlier studies (Kuznetsov *et al.*, 2002 ▸; Miller & Schuele, 1969 ▸; Waldorf & Alers, 1962 ▸).

### 2.7. PIXEL, Hirshfeld surface and symmetry-adapted perturbation theory calculations   

Molecular electron densities were obtained using the program *GAUSSIAN09* (Frisch *et al.*, 2016 ▸) at the MP2 level of theory with the 6-31G** basis set. The electron density was then passed to the *PIXELC* module of the *CLP* program package, allowing the calculation of dimer and lattice energies (Gavezzotti, 2005 ▸, 2007 ▸, 2011 ▸). Hirshfeld surface calculations were carried out with *CrystalExplorer* (Turner *et al.*, 2017 ▸) using the same level of theory and basis set as the PIXEL calculations. Symmetry-adapted perturbation theory calculations were carried out with the PSI-4 code (version Beta5) using the SAPT2+3 method (Hohenstein & Sherrill, 2010*a* ▸,*b* ▸) with the aug-cc-pVDZ basis set. δ*E*_HF_ (Gonze *et al.*, 1994 ▸) corrections were applied to induction energies in all cases. In each case the C—H distances were reset to 1.089 Å to correct for the systematic shortening of bonds to hydrogen in crystal structures determined from X-ray data.

### 2.8. Periodic density functional theory (DFT) calculations   

Geometry optimizations were carried out using the plane-wave pseudopotential method in the *CASTEP* code (Clark *et al.*, 2005 ▸) as incorporated into *Materials Studio* (Dassault Systèmes BIOVIA, 2017 ▸). The PBE exchange-correlation functional was used with norm-conserving pseudopotentials and a basis set cut-off energy of 920 eV (Perdew *et al.*, 1996 ▸). Brillouin zone integrations were performed with a Monkhorst–Pack **k**-point grid spacing of 0.07 Å^−1^ (Monkhorst & Pack, 1976 ▸). The starting coordinates were taken from the single-crystal diffraction studies of Sections 2.1 and 2.2 and optimized using the Tkatchenko–Scheffler correction for dispersion (DFT-D) (Tkatchenko & Scheffler, 2009 ▸). The cell dimensions were fixed to the experimental values, and the space group symmetry was retained. The energy convergence criterion was 1 × 10^−8^ eV per atom, with a maximum force tolerance of 0.001 eV Å^−1^ and a maximum displacement of 1 × 10^−5^ Å; the SCF convergence criterion was 1 × 10^−10^ eV per atom. Frequencies were calculated (Refson *et al.*, 2006 ▸) at the Γ-point only for the h_5_ isotopologues as the results were intended for comparison with the experimental Raman spectra described in Sections 2.4 and 2.5.

### 2.9. Symmetry-mode analysis with *ISODISTORT*   

The *ISODISTORT* software (Campbell *et al.*, 2006 ▸) from the *ISOTROPY* software suite (https://iso.byu.edu) was used to decompose the pattern of molecular translations and rotations that arise in the phase III→II transition into symmetry modes of the phase III space group (*P*4_1_2_1_2). By symmetry modes, we mean basis functions of the irreducible matrix representations of the parent symmetry group, which are patterns that transform under the influence of each symmetry operation according to the coefficients within the corresponding representation matrix. In general, one can always parameterize the symmetry-breaking order parameters of a phase transition into symmetry modes of the parent symmetry group.

The amplitude of a mode is the root-summed-squared magnitude of the atomistic changes affected by the mode over the primitive unit cell of the low-symmetry phase (Å for displacements, radians for rotations). A symmetry-mode vector in crystal-axis coordinates is obtained by multiplying its simplified form (*e.g.* 110) by the corresponding amplitude and normalization factor. The normalization factor ensures that the root-summed-squared magnitude will be 1 when the amplitude has a value of 1. In the present case, where this sum runs over the four centroid positions of *Z* = 4 copies of a single symmetry-unique molecule in the unit cell, a molecular displacement or rotation angle can be simply computed by dividing the corresponding mode amplitude by 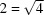.

In a phase transition that includes lattice strain, molecular motions can arise separately from displacive/rotational symmetry modes and from the lattice strain itself (assuming an inherently irrotational strain). The displacive and rotational symmetry modes in *ISODISTORT* are defined purely in terms of the unstrained unit cell, and only contribute to the rotations that are not geometrically required by the changing shape of the unit cell. During lattice strain, fixing a symmetry-mode amplitude fixes the lattice-coordinate components (unitless for displacements, radians Å^−1^ for rotations) of the corresponding atomic displacement and rotation vectors, though the Cartesian components (Å for displacements, radians for rotations) may vary slightly with the strain at fixed-mode amplitude.

## 3. Results and discussion   

### 3.1. Formation of pyridine III   

Prior to this work pyridine was acknowledged to form crystalline phases I (*Pna*2_1_ *Z*′ = 4) and II (*P*2_1_2_1_2_1_ *Z*′ = 1) as well as an amorphous form (Castellucci *et al.*, 1969 ▸; Fanetti *et al.*, 2011 ▸; Heyns & Venter, 1985 ▸; Mootz & Wussow, 1981 ▸; Podsiadło *et al.*, 2010 ▸; Zhuravlev *et al.*, 2010 ▸). The present results yield the structural and spectroscopic characterization of a third crystalline form, designated phase III (*P*4_1_2_1_2, *Z*′ = ½). The Pearson symbols for these phases are *oP*16, *oP*4 and *tP*4. Crystals of phases II and III were grown from pure pyridine-h_5_ by *in situ* crystallization at 1.09 and 1.69 GPa, respectively, using the ‘approximately isochoric’ procedure described in Sections 1 and 2. Phase III can also be obtained by crystallization of pyridine from a solution in methanol, though this procedure can also lead to the formation of a methanol solvate (Podsiadło *et al.*, 2010 ▸).

Dunitz & Schweizer (2006 ▸, 2007 ▸) have drawn attention to the tendency for approximately hexagonal molecules with *C*_2v_ point symmetry to crystallize in the space group *P*4_1_2_1_2 (or equivalently *P*4_3_2_1_2) with unit-cell dimensions in the region of *a* = 6 and *c* = 14 Å. Examples include alloxan (*a* = 5.84, *c* = 13.85 Å) and fluoro­benzene (*a* = 5.80 and *c* = 14.51 Å). Pyridine III conforms to this pattern, with *a* = 5.4053 (4) and *c* = 13.44853 (14) Å at 1.69 GPa. The X-ray powder pattern for a polymorph of benzene that exists between 1.4 and 4 GPa (Thiéry & Léger, 1988 ▸), which seems somewhat experimentally elusive, but is discussed in computational studies (van Eijck *et al.*, 1998 ▸; Wen *et al.*, 2011 ▸), can be indexed with a tetragonal unit cell of dimensions *a* = 5.29 and *c* = 14.29 Å at 3.1 GPa (van Eijck *et al.*, 1998 ▸).

The unusual characteristics of crystalline pyridine have inspired a number of crystal structure prediction studies (Aina *et al.*, 2017 ▸; Anghel *et al.*, 2002 ▸; Gavezzotti, 2003 ▸; van de Streek & Neumann, 2011 ▸). In the most recent of these, Aina *et al.* (2017 ▸) generated a force field that was trained using energies calculated with symmetry-adapted perturbation theory (SAPT) for gas-phase dimers. The results enabled them to identify the structure of phase III reported here in an energy landscape calculated at 2 GPa.

### 3.2. The crystal structure of pyridine III and its relationship with pyridine II   

In phase III the twofold axis of the pyridine molecule is constrained to lie along the twofold axis of the space group (*P*4_1_2_1_2), and only the orientation of the molecule about this axis is unconstrained by symmetry. Relative to a Cartesian model with the twofold axis along *z* and the molecule in the *xz* plane, the orientation in the unit cell is obtained by rotations of 90, 136.4 and −45° about *a*, *b* and *c*. Pyridine II crystallizes in *P*2_1_2_1_2_1_, which is a subgroup of *P*4_1_2_1_2, with cell dimensions which are similar to phase III. The molecular orientation parameters at 1.09 GPa are also similar to those in phase III: 92.2, 127.6 and −47.2°. The rings in phase II are more perpendicular to the *c* axis, which is therefore shorter than in phase III. There is no suggestion that pressure distorts the intramolecular geometry, as was the case in *syn*-1,6:8,13-bis­carbonyl­[14]annulene (Casati *et al.*, 2016 ▸).

An analysis of changes in the Cartesian-coordinate atom positions, calculated using the actual cell parameters of each phase, shows that the pyridine molecule rotation angle is 9.5° around an axis that is roughly 25° from the N1—C4 axis and 11° out of the plane of the molecule. If the lattice strain (*i.e.* the change in the cell dimensions over the transition from phase III to II) is neglected by using the phase III cell parameters to calculate the Cartesian coordinates of both phases, the pyridine rotation angle increases to 19.6° along roughly the same direction. But if instead the changes to the internal lattice coordinates of each atom are neglected by considering only the strain-induced changes to the Cartesian coordinates, the pyridine rotation angle drops back to 8.9° but reverses its direction. Thus, the internal and strain-induced contributions act in nearly opposite directions, so that roughly half of the internal rotation exists to maintain a regular internal geometry and optimize intermolecular interactions throughout the strain.

Topological analysis indicates that phase II has 14 molecules in its first coordination sphere with 50 and 110 molecules in the second and third coordination spheres, respectively [calculated using *ToposPro*, (Blatov *et al.*, 2014 ▸)]. This ‘14–50–110’ coordination sequence is characteristic of a body-centred cubic packing topology (*bcu-x* in the notation of the RCSR topological database; Peresypkina & Blatov, 2000 ▸; O’Keeffe *et al.*, 2008 ▸). Phase III is characterized by a coordination sequence of 12–42–92, which corresponds to face-centred cubic close packing (RCSR symbol *fcu*), indicating that the effect of higher pressure is to eject two molecules from the coordination sphere of phase II to generate a more close-packed topology.

Intermolecular interaction energies were calculated using the PIXEL method and symmetry-adapted perturbation theory at the SAPT2+3 level (Tables 2 and 3). The total energies obtained in the two sets of calculations are in excellent agreement, with the maximum difference being 1.3 kJ mol^−1^ for a CH⋯π contact in phase III. Aina *et al.* (2017 ▸) identified eight pyridine dimers as potential energy minima in the gas phase. The dimers are linked by CH⋯N, CH⋯π or stacking interactions and have energies in the range from −14.31 to −16.56 kJ mol^−1^. These energies are systematically more negative (*i.e.* more stabilizing) than those listed in Tables 2 and 3 because they represent optimized potential energy minima for isolated dimers. Both PIXEL and SAPT methods break down the total energies into electrostatic, polarization, dispersion and repulsion terms, and although there is more variation between PIXEL and SAPT in the component energies than in the total energies, it is clear from both calculations that dispersion is the dominant term in all contacts. This finding is in agreement with Aina *et al.*’s and Gavezzotti’s previous results (Aina *et al.*, 2017 ▸; Gavezzotti, 2003 ▸).

**Figure 2:**
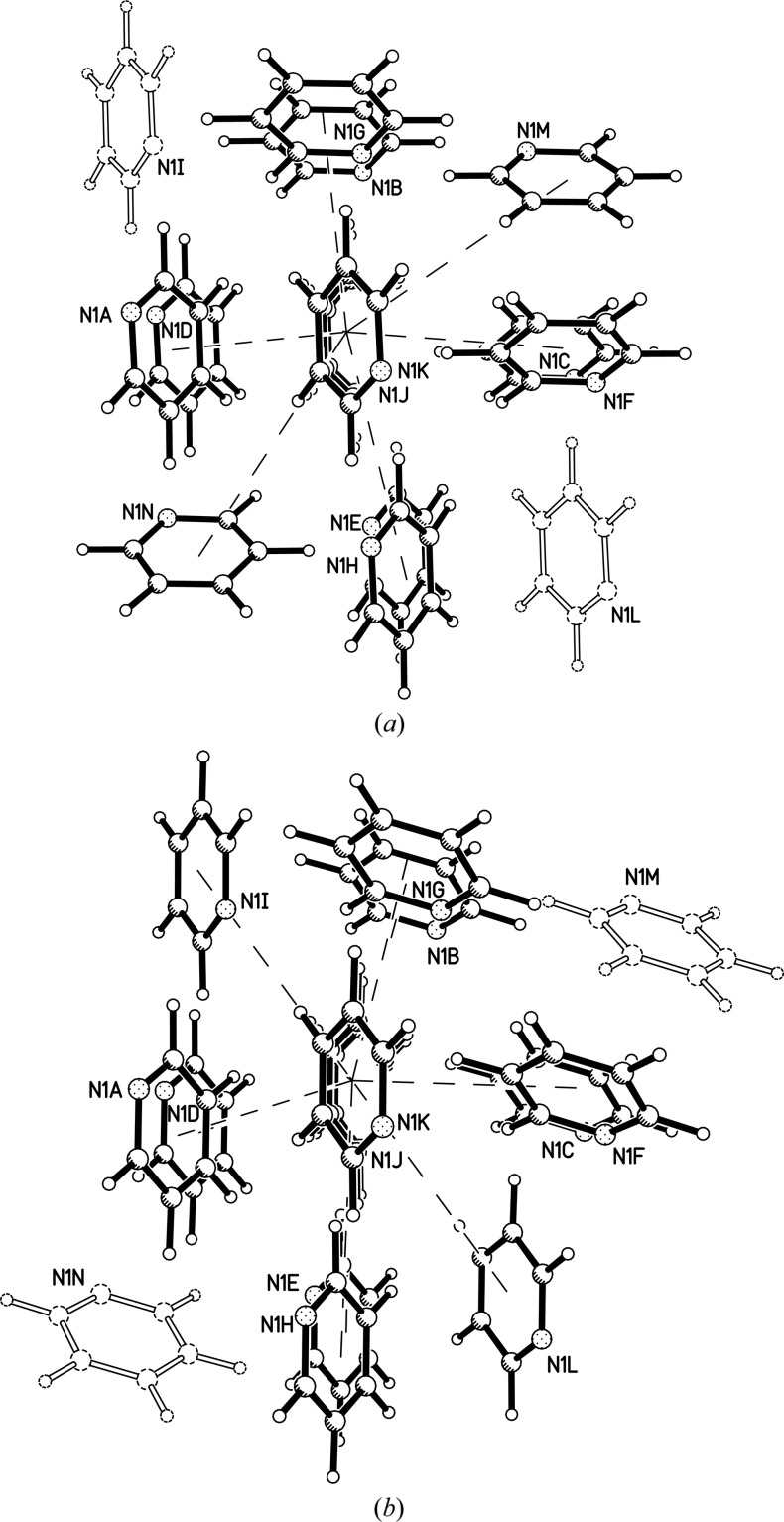
First coordination spheres of (*a*) pyridine II at 1.09 GPa and (*b*) pyridine III at 1.69 GPa. The molecules described in the text as forming long interactions are shown as outlines.

The first coordination spheres of both phases II and III contain 12 molecules with centroid–centroid distances between *ca* 4.5 and 6 Å. An additional pair of contacts form at distances of 6.8 Å in phase II and 7.1 Å in phase III. The increase in these longer contact distances for phase III is the reason the packing topology changes from *bcu* to *fcu* (see above). Equivalent contacts in the two phases are correlated in Figs. 2 ▸(*a*) and 2(*b*) and Tables 2 ▸ and 3 ▸, where molecules in related positions in the two phases carry the same label. In Fig. 2 ▸(*b*) (phase III) the 12 contacts between 4.5 and 6 Å are labelled A–L, with the two longer (6.8 Å) interactions labelled M and N. In phase II [Fig. 2 ▸(*a*)] the contacts to molecules I and L lengthen as molecules M and N move into the first coordination sphere. Animations of the transition based on the symmetry-mode analysis in Section 3.5 are given in the supporting information, which also contains plots of the individual dimers (Fig. S5).

The total lattice energy of phase II at 1.09 GPa is calculated by the PIXEL method to be −60.0 kJ mol^−1^, and that of phase III at 1.69 GPa to be −56.3 kJ mol^−1^. These values can be compared with values of −64.94, −66.76 and −65.36 kJ mol^−1^, respectively, calculated for the structures of phases I, II and III optimized at ambient pressure (Aina *et al.*, 2017 ▸). The 12 strongest contacts comprise six pairs of symmetry-related contacts in phase II and three sets of four in phase III. The molecule–molecule energies range between −12.4 and −4.6 kJ mol^−1^ in phase II and −9.0 and −7.1 kJ mol^−1^ in phase III (SAPT values). Three classes of contacts are formed.

**Figure 3:**
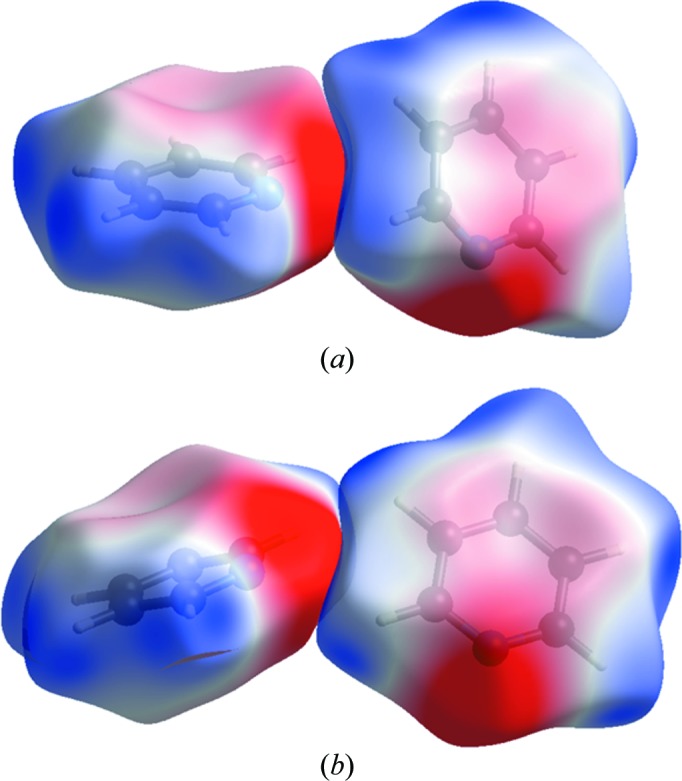
CH⋯N contacts in (*a*) phase II and (*b*) phase III, depicted using Hirshfeld surfaces coloured according to electrostatic potential. Note the better alignment of the blue (positive) and red (negative) regions of the potentials in phase II. The potentials are mapped over the range ±0.05 a.u.

One class (contacts E–H) is characterized by intermolecular CH⋯N interactions with H⋯N distances of between 2.58 and 2.76 Å and centroid–centroid distances near 6 Å. Contacts E–H are all related by symmetry in phase III (C2H2⋯N1 = 2.69 Å, ∠C2H2⋯N1 = 142°, −7.6 kJ mol^−1^), but split in phase II into two sets of two contacts E/H (C2H2⋯N1 = 2.58 Å, ∠C2H2⋯N1 = 135°, −10.8 kJ mol^−1^) and F/G (C2H2⋯N1 = 2.76 Å, ∠C2H2⋯N1 = 124°, −12.4 kJ mol^−1^). Although dispersion is the largest term, changes in the coulombic contributions determine the energy differences between phases II and III, as a result of the more optimal alignment of positive and negative regions of the electrostatic potentials of the interacting molecules in phase II. This is illustrated using Hirshfeld surfaces coloured according to the molecular electrostatic potentials in Fig. 3 ▸ (Spackman *et al.*, 2008 ▸; Spackman & Jayatilaka, 2009 ▸). In general, high-pressure crystal structures can accommodate (energetically) weaker intermolecular interactions by minimizing the *PV* contribution to free energy through more efficient packing (see Section 3.3).

A second set of contacts (A–D) consists of CH⋯π interactions with centroid–centroid distances of around 4.5 Å, CH⋯ring centroid distances of between 2.57 and 3.12 Å, and energies between −9 and −10 kJ mol^−1^. The magnitudes of the coulombic energies in these contacts are only about 20–30% of the dispersion energies. A third set of interactions (I–L in phase III and J, K, M and N in phase II) with centroid–centroid distances of 5.39–5.86 Å, are dominated by dispersion with very little coulombic energy and a lack any distinctive atom–atom contacts. Note that π⋯π contacts do not occur in any of the phases.

Both phases I and II contain CH⋯π and CH⋯N contacts, but over the course of the I→II transformation some CH⋯π contacts are converted into CH⋯N interactions as a result of molecular reorientations. By contrast, the correlations shown in the animations (see supporting information) and in Tables 2 ▸ and 3 ▸ show that nature of each interaction remains constant over the course of the transition between phases II and III, so that CH⋯N contacts in one phase transform in to CH⋯N contacts in the other *etc*., but the distances and energies change. The lack of a characteristic geometric signature for contacts I–L gives them a degree of flexibility, allowing them to accommodate the biggest changes during the transition.

### 3.3. Equations of state of pyridine II and pyridine III   

**Figure 4:**
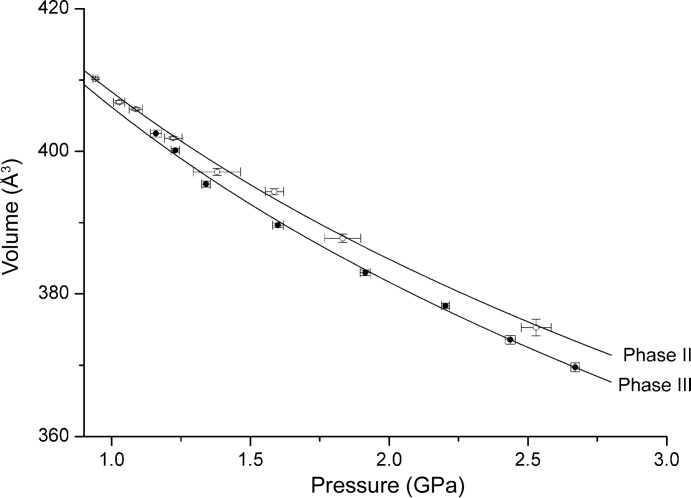
Variation of the unit-cell volumes of pyridine-d_5_ phases II (open circles) and III (closed circles) with pressure, fitted to the third-order Birch–Murnaghan equation of state.

The variations of unit-cell volume with pressure for pyridine II and III, as determined using neutron powder diffraction, were fitted to the third-order Birch–Murnaghan equation of state (Fig. 4 ▸) using the EoSFIT7 code (Gonzalez-Platas *et al.*, 2016 ▸). The number of points obtained is quite limited, with pressures starting at *ca* 1 GPa, and it was not possible to refine values of the zero-pressure cell volume (*V*_0_), the bulk modulus (*K*_0_) and its pressure derivative (*K*′) simultaneously. *V*_0_ was therefore fixed at 452 Å^3^, the volume of pyridine II at 195 K. *K*_0_ for phase II was found to be 6.4 (3) GPa with *K*′ = 8.7 (10). Corresponding figures for phase III are 6.2 (3) GPa and 8.1 (9). The two sets of parameters are the same within their uncertainties. The consistently lower volume of phase III is consistent with it becoming the more stable phase at elevated pressure, whereas its higher density is consistent with a more close-packed topology.

A small bulk modulus (<10 GPa) is typical of soft materials where dispersion forces dominate intermolecular interactions, *e.g.* naphthalene and Ru_3_(CO)_12_ have values of 8.3 (4) and 6.6 GPa, respectively (Likhacheva *et al.*, 2014 ▸; Slebodnick *et al.*, 2004 ▸). Flexible intramolecular torsion angles can provide an additional mechanism for compression, as seen in the P and OP polymorphs of the prodigiously polymorphic compound ‘ROY’ {5-methyl-2-[(2-nitro­phenyl)­amino]-3-thio­phene­carbo­nitrile}, for which *K*_0_ = 6.0 (7) and 4.3 (3) GPa, respectively (Harty *et al.*, 2015 ▸; Funnell *et al.*, 2019 ▸). It assumes a higher value if additional intermolecular interactions such as hydrogen bonding are present, *e.g.* the values for hydro­quinone–formic acid clathrate, melamine and l-alanine are 13.6 (4) GPa (Eikeland *et al.*, 2016 ▸), 12.0 (5) GPa (Fortes *et al.*, 2019 ▸) and 13.4 (7) GPa (Funnell *et al.*, 2010 ▸), respectively. Materials characterized by a mixture of dispersion and weaker hydrogen bonds have correspondingly lower bulk moduli, *e.g.* aniline (phase II) is 5.4 (2) (Funnell *et al.*, 2013 ▸).

### 3.4. Raman spectra   

**Figure 5:**
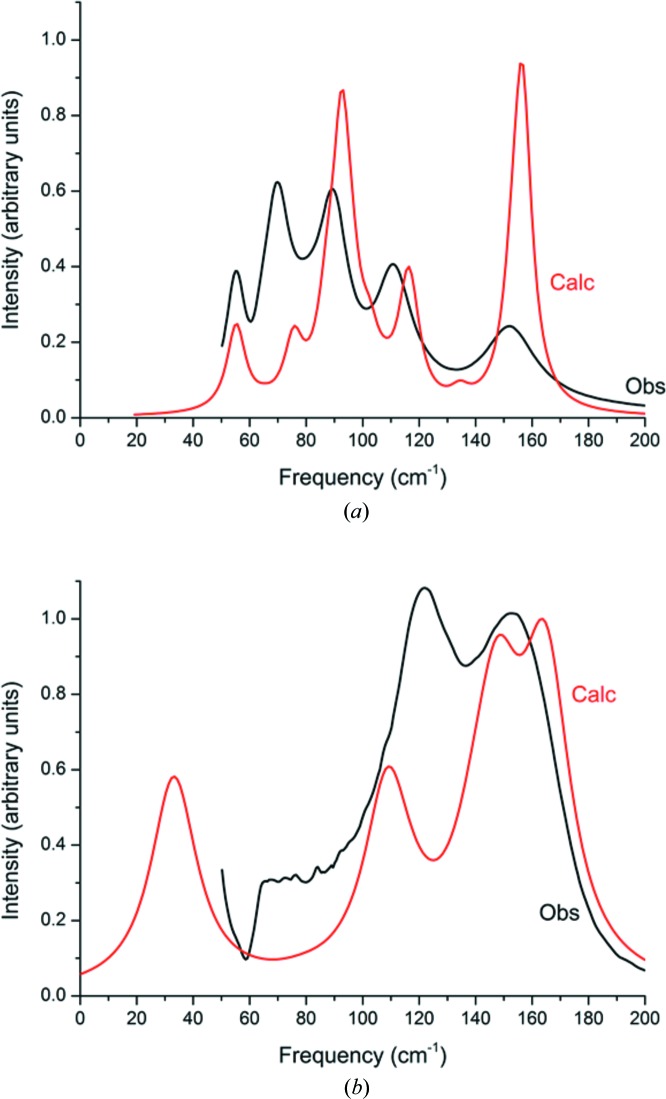
The Raman spectra in the lattice phonon region of (*a*) phase II at 1.09 GPa and (*b*) phase III at 1.69 GPa, collected in the same regions as the diffraction data. Observed data – black; DFT-calculated data – red.

The inconsistencies in the literature regarding the assignment of Raman spectra of the different high-pressure forms of pyridine were described in Section 1. With the aim of resolving the ambiguities, Raman spectra were measured on the same samples of phases II and III as had been used for X-ray data collections. The regions of the spectra below 200 cm^−1^, which contain the external or ‘lattice’ modes, are shown in Fig. 5 ▸. The spectra are compared with each other and with those found in the literature in Table 4 ▸.

The Raman spectrum of phase II (*P*2_1_2_1_2_1_) has five bands below 200 cm^−1^, matching the spectrum collected at 1 GPa by Heyns & Venter (1985 ▸), shown at the far left of Fig. 1 in their paper. The spectrum of phase III (*P*4_1_2_1_2) is quite different, with two distinct bands matching the (quite broad) spectra measured at above 1.7 GPa by Fanetti *et al.* (2011 ▸), at 2 GPa by Zhuravlev *et al.* (2010 ▸) and at 2.5 GPa by Heyns & Venter (1985 ▸) (far-right spectrum of Fig. 1 in their paper). The band below 50 cm^−1^ is discussed in Section 3.5.

The frequencies of the Raman modes observed for phases II and III are reproduced to within 10 cm^−1^ in periodic DFT calculations without any need for frequency scaling. The intensities of the bands are more poorly reproduced; discrepancies between calculated and experimental Raman intensities have been noted previously, *e.g.* for tetracene (Abdulla *et al.*, 2015 ▸). The calculations enable the modes to be assigned to oscillations about axes lying in the planes of the pyridine molecules.

It is clear from these data that the ‘phase I’ of Heyns & Venter (1985 ▸) is actually the *Z*′ = 1, *P*2_1_2_1_2_1_ phase II, and the ‘phase II’ of Fanetti *et al.* (2011 ▸) and Zhuravlev *et al.* (2010 ▸) is the *Z*′ = ½, *P*4_1_2_1_2 phase III. Interestingly, the spectrum of pyridine-h_5_ recorded at 2.5 GPa by Heyns & Venter contains additional bands between 57 and 108 cm^−1^, which are neither seen in Fanetti’s or Zhuravlev’s spectra, nor by us in our experimental or calculated spectra. It is anomalous that an increase in crystallographic symmetry should give rise to a more complex Raman spectrum, and it seems possible that their sample was contaminated with phase II.

### 3.5. Decompression of pyridine III and symmetry mode analysis   

The unit cells of phase II and III characterize similar translational symmetry, but the structures differ in the relative orientations of the molecules (Section 3.2); the transition between phases III and II therefore occurs at the Γ-point of reciprocal space. Symmetry mode analysis (*ISODISTORT*) indicates that the motions affected by the transition can be concisely described in terms of molecular rotations and translations belonging to the totally symmetric Γ_1_ (*A*_1_ in Mulliken notation) and the Γ_2_ irreducible representations. The Γ_2_ mode could correspond to either of the Mulliken symbols *B*_1_ or *B*_2_ depending on twofold axis definitions; *B*_2_ will be used here. The *A*_1_ mode rotates the pyridine molecules about their twofold axes, which does not lower the space group symmetry. The *B*_2_ mode causes the twofold molecular axes to deviate from the crystallographic twofold axes (see Section 3.2), thereby reducing the space group symmetry of the crystal from *P*4_1_2_1_2 to *P*2_1_2_1_2_1_ [note that the standard settings of these space groups also require an origin shift of (1/4, 0, 3/8)].

Each *A*_1_ and *B*_2_ mode is capable of molecular translations parallel or antiparallel to the affected rotations. *A*_1_ is responsible for most of the molecular translation, while *B*_2_ is responsible for approximately half the molecular rotation. The intrinsic rotational symmetry modes and their amplitudes are shown in Table 5 ▸, which are defined so as not to include the rather significant strain-induced rotations; the overall strain-adjusted rotation angle is roughly half the value that can be inferred from the table (see Section 3.2). The root-sum-squared aggregate displacive amplitudes are 0.765 Å for Γ_1_ and 0.147 Å for Γ_2_, which yields an overall displacive amplitude of 0.779 Å. Similarly, the root-sum-squared aggregate rotational amplitudes are 0.550 radians for Γ_1_ and 0.417 radians for Γ_2_, which yields an overall rotational amplitude of 0.690 radians. Details of the methods used to calculate the data in Table 5 ▸ will be published separately.

The lowest Γ-point vibrational frequencies in pyridine III have the same *A*_1_ and *B*_2_ symmetries that govern the movements and rotations of the molecules through the phase III→II transition. The calculations of Fig. 5 ▸ indicate that the *A*_1_ vibrational mode can be observed by Raman spectroscopy in phase III at 33 cm^−1^. The *B*_2_ mode (calc. 52 cm^−1^), though formally Raman active, is predicted to have zero intensity. Neither mode is observable by infra-red spectroscopy.

**Figure 6:**
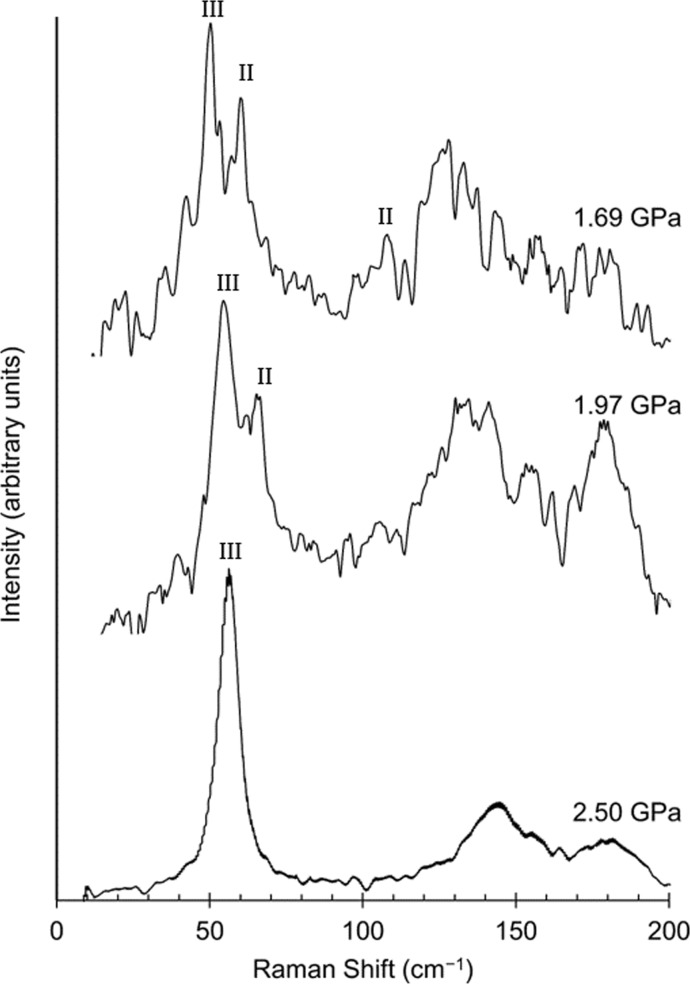
Raman spectra of the decompression of pyridine III to a pyridine III/II mixture.

A single crystal of pyridine III was grown by high-pressure *in situ* crystal growth from methanol and Raman measurements were performed in the region below 200 cm^−1^ on decompression from 2.50 to 1.69 GPa (Fig. 6 ▸). All of the spectra have a high luminescence background, which becomes more prominent on decreasing pressure and is still present after background corrections and spectral averaging. The spectrum collected at 2.50 GPa is that of pyridine III. There is a single strong band at 57 cm^−1^, which, on the basis of the DFT calculations, is assigned to the *A*_1_ mode described above. The frequency is somewhat higher than had been calculated for the structure at 1.69 GPa (33 cm^−1^, Table 4 ▸), but if the calculation is repeated with the cell dimensions measured at 2.67 GPa the agreement is much better (60 cm^−1^).

The onset of the pyridine III→II phase transition can been seen on decompression from 2.50 to 1.97 GPa from the emergence of the phase II band at 71 cm^−1^ (*cf.* Table 4 ▸). The intensity of this band increases at 1.69 GPa, with an additional band beginning to grow-in at 100 cm^−1^ also being consistent with the formation of phase II. Further decompression (to 1.55 GPa) led to dissolution of the pyridine in the methanol and a noisy luminescence spectrum from which no Raman data could be extracted. These data show that the transition between phases III and II does not occur sharply, but gradually occurs as pressure is reduced, an observation that is consistent with the sluggishness of the transition between phases I and II in pyridine-d_5_ as a function of temperature.

**Figure 7:**
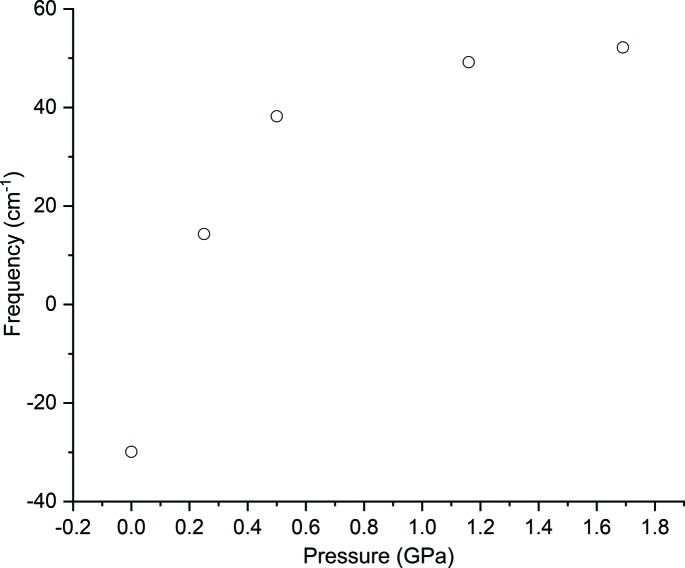
Variation of the frequency of the of the B_2_ symmetry-breaking lattice mode as a function of pressure. Frequencies were calculated by periodic DFT; imaginary frequencies are shown as negative numbers.

The symmetry-mode analysis suggests that the *B*_2_ vibration is expected to act as a soft mode. Since it is not observable by optical spectroscopy, its frequency was calculated using periodic DFT using cell dimensions observed for phase III at selected pressures, with three extra points at 0, 0.25 and 0.50 GPa inferred from the equation of state. The variation (Fig. 7 ▸) shows a rapid softening of the mode as pressure is reduced. The transition pressure was calculated to occur at about 1 GPa lower than observed experimentally. The difference may of course be related to approximations in the calculations, for example, the assumption of harmonic behaviour. Alternatively, it may imply that the transition initiates at crystal defects, where the local pressure would be expected to be lower, and proceeds via the nucleation and growth mechanism usually associated with reconstructive transitions.

## 4. Conclusions   

The Cambridge Structural Database (Groom *et al.*, 2016 ▸) (established 1965) has just celebrated its one-millionth entry. Of these about 5000 structures resemble pyridine I in having four molecules in the asymmetric unit. Only around 1200 have *Z*′ greater than this. Steed & Steed (2015 ▸) have identified a number of factors that may promote formation of crystal structures with more than one molecule in the asymmetric unit. While the phenomenon is slightly more common in small molecules than in larger ones (Anderson *et al.*, 2011 ▸; Gavezzotti, 2008 ▸), in the case of pyridine I it appears that the reason lies in the energetic competitiveness of CH⋯N and CH⋯π interactions (Aina *et al.*, 2017 ▸; Crawford *et al.*, 2009 ▸). Chains of molecules in pyridine I are formed by a mixture of these interactions (Crawford *et al.*, 2009 ▸). A small amount of pressure, of the order of 1 GPa, is enough to convert some of the CH⋯π into CH⋯N contacts, forming *Z*′ = 1 phase II.

Phase II has a lower molar volume than phase I and the transition between the two is driven by the influence of the pressure × volume contribution to free energy, which favours efficient packing. A similar feature was observed in methyl 2-(carbazol-9-yl)benzoate, where a *Z*′ = 8 ambient pressure form converts to a *Z*′ = 2 form at 5.3 GPa by selection of lower-volume molecular conformations (Johnstone *et al.*, 2010 ▸). In iso­propanol the same factor applies, but in this case it leads to an increase in *Z*′ from 3 to 4 at 1.1 GPa as the structure sacrifices hydrogen-bond linearity to improve packing efficiency (Ridout & Probert, 2014 ▸).

Increasing the crystallization pressure from 1.1 to 1.7 GPa leads to the formation of pyridine III. The underlying close-packed topology of this polymorph gives it an even lower volume than pyridine II, which has a body-centred cubic topology. Fanetti *et al.*’s (2011 ▸) Raman data show that phase III persists all the way to 18 GPa; the additional phases proposed by Zhuravlev *et al.* (2010 ▸) were not reproduced in Fanetti’s work.

In the light of our combined crystallographic and Raman data, Heyns & Venter’s, Fanetti *et al.*’s and Zhuravlev *et al.*’s phases I and II are phases II and III, respectively. Phase I does not feature in any of these papers.

There is no rational geometric relationship between the crystal structures of pyridine I and II, and there is no group–subgroup relationship between their space groups (*Pna*2_1_ and *P*2_1_2_1_2_1_). The transition between the two can be studied thermally provided the perdeuterated isotopologue is used, but the transition causes a single crystal of phase I to break into fragments. The only crystallographically verified literature report of phase I being formed at high pressure is in Podsiadło’s study, in which it was formed at room temperature on increasing pressure on the liquid from 1.0 and 1.2 GPa (Podsiadło *et al.*, 2010 ▸). Increasing the pressure on this sample to 2 GPa caused it to break up. It was assumed at the time that phase II had formed, but the pressure implies that it could have been phase III.

Phases II and III are geometrically much more closely related than phases I and II, and a group–subgroup relationship exists between their space groups (*P*2_1_2_1_2_1_ and *P*4_1_2_1_2). The transition between phases III and II is displacive and governed by mode-softening. The soft mode cannot be observed experimentally by Raman or infra-red spectroscopy, but it was inferred by a combination of symmetry mode analysis of the crystal structures and periodic DFT calculations. However, the transition is not concerted, and it may proceed via nucleation at defects.

The transition between phases III and II can be reproducibly observed by releasing pressure: in this work its onset was seen as pressure was reduced from 2.5 to 1.97 GPa (Fig. 6 ▸), in Fanetti’s work it was seen at 1 GPa. The expected transition from phase II to III on pressure increase is less reproducibly observed, but both Heyns & Venter and Zhuravlev *et al.* made this observation. However, Fanetti *et al.* were able to compress phase II to 9.4 GPa, whereas in this work we compressed a sample of the deuterated isotopologue to 2.5 GPa, albeit with some reversible amorphization.

The variability of the transition pressures and sequences quoted above indicate that kinetics are an important factor in the phase behaviour of pyridine, and authors frequently remark on the sluggishness of its phase transitions. This even extends to the melting transition. Although the melting pressure of pyridine is 0.55 GPa, crystallization is only ever observed at around 1 GPa or above (Fanetti *et al.*, 2011 ▸; Podsiadło *et al.*, 2010 ▸). Crystal growth often has to be induced by increasing pressure in small increments or with thermal annealing, and pyridine is highly susceptible to the formation of glassy or amorphous phases. Fanetti’s paper illustrates featureless Raman spectra up to 1.6 GPa, while we failed to see any evidence of crystalline diffraction at pressures as high as 2.7 GPa, the sample only crystallizing on release of pressure. Similar features characterize the phase behaviour of benzene (Thiéry & Léger, 1988 ▸; Podsiadło *et al.*, 2010 ▸; Chanyshev *et al.*, 2018 ▸; Cansell *et al.*, 1993 ▸).

To summarize, phases II and III can both be accessed reproducibly by crystal growth directly from the liquid phase. Phase II can also be accessed by decompression of phase III or from decompression of a pressure-amorphized sample. The sequence of phases I→II→III has never been observed at high pressure in a single sample.

## Supplementary Material

Crystal structure: contains datablock(s) pyridine2_final, pyridine3_final. DOI: 10.1107/S2052252519015616/lq5025sup1.cif

Animation of the transition viewed along a. DOI: 10.1107/S2052252519015616/lq5025sup2.gif

Animation of the transition viewed along b. DOI: 10.1107/S2052252519015616/lq5025sup3.gif

Animation of the transition viewed along c. DOI: 10.1107/S2052252519015616/lq5025sup4.gif

Raman spectra (Figs. S1-S4) and intermolecular contacts (S5). DOI: 10.1107/S2052252519015616/lq5025sup5.pdf

Video of the transition viewed along a. DOI: 10.1107/S2052252519015616/lq5025sup6.mp4

Video of the transition viewed along b. DOI: 10.1107/S2052252519015616/lq5025sup7.mp4

Vidoe of the transition viewed along c. DOI: 10.1107/S2052252519015616/lq5025sup8.mp4

Raman spectra (Figs. S1-S4) and intermolecular contacts (S5). DOI: 10.1107/S2052252519015616/lq5025sup5.pdf

CCDC references: 1966460, 1966461, 1966461

## Figures and Tables

**Table 1:** Crystal and refinement data For all structures: C_5_H_5_N, *M*_r_ = 79.10. Experiments were carried out at 293 K with synchrotron radiation, λ = 0.49594 Å using a Perkin–Elmer a-Si detector. H atom parameters were constrained. Absolute structure parameters are inconclusive.

	Pyridine II at 1.09 GPa	Pyridine III at 1.69 GPa
Crystal data		
Crystal system, space group	Orthorhombic, *P*2_1_2_1_2_1_	Tetragonal, *P*4_1_2_1_2
*a*, *b*, *c* (Å)	5.392 (3), 6.806 (3),11.261 (5)	5.4053 (4), 5.4053 (4),13.4853 (14)
α, β, γ (°)	90, 90, 90	90, 90, 90
*V* (Å^3^)	413.2 (3)	394.00 (7)
*Z*, *Z*′	4, 1	4, ½
μ (mm^−1^)	0.04	0.04
Crystal size (mm)	0.26 × 0.26 × 0.1	0.35 × 0.35 × 0.1

Data collection		
Absorption correction	Multi-scan	Multi-scan
*T*_min_, *T*_max_	0.484, 0.744	0.585, 0.745
No. of measured, independent and observed [*I* > 2σ(*I*)] reflections	1324, 411, 291	1328, 326, 297
*R*_int_	0.033	0.061
(sin θ/λ)_max_ (Å^−1^)	0.625	0.625

Refinement		
*R*[*F* ^2^> 2σ(*F* ^2^)], *wR*(*F* ^2^), *S*	0.029, 0.060, 0.88	0.076, 0.196, 1.14
No. of parameters	56	30
Completeness (%)	52	77
Δρ_max_, Δρ_min_ (e Å^−3^)	0.04, −0.05	0.25, −0.30
Absolute structure parameter	10.0 (10)	−10.0 (10)

**Table 2:** Intermolecular contacts in pyridine III. All energies are in kJ mol^−1^ and contact distances (in Å) were calculated with C—H distances reset to 1.089 Å

Label	Centroid distance	Symmetry		Coulombic energy	Polarization energy	Dispersion energy	Repulsion energy	Total energy	Contacts
A	4.466	*x* + ½, −*y* + ½, −*z* + 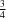	PIXEL	−5.3	−3.5	−18.2	19.3	−7.7	C3H3⋯π = 2.61 Å (∠ = 129°)
B		−*x* + ½, *y* − ½, −*z* + 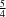	SAPT2+3	−8.4	−3.2	−23.7	26.2	−9.0	
C		−*x* + ½, *y* + ½, −*z* + 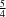							
D		*x* − ½, −*y* + ½, −*z* + 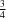							
									
E	6.094	*x* − ½, −*y* + 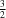 , −*z* + 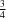	PIXEL	−5.6	−3.8	−12.0	13.1	−8.3	C2H2⋯N1 = 2.69 Å (∠ = 142°)
F		−*x* + 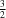 , *y* + ½, −*z* + 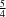	SAPT2+3	−6.8	−3.0	−13.8	16.0	−7.6	
G		−x + 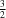 , y − ½, −z + 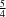							
H		*x* + ½, −*y* + 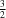 , −*z* + 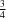							
									
I	5.410	*x*, *y* − 1, *z*	PIXEL	−2.2	−1.5	−10.0	6.9	−6.9	Non-specific dispersion: highly slipped stack, β = 61°. Interplanar distance = 2.64 Å
J		*x* + 1, *y*, *z*	SAPT2+3	−4.7	−1.9	−13.4	12.9	−7.1	
K		*x* − 1, *y*, *z*							
L		*x*, *y* + 1, *z*							
									
M	7.111	−*x* + 1, −*y* + 1, *z* + ½	PIXEL	0.0	−0.1	−1.0	0.0	−1.0	Long-range dispersion, H⋯H = 4.23 Å
N		−*x* + 1, −*y* + 1, *z* − ½	SAPT2+3	0.1	−0.1	−1.5	0.1	−1.4	

**Table 3:** Intermolecular contacts in pyridine II. All energies are in kJ mol^−1^ and contact distances (in Å) were calculated with C—H distances reset to 1.089 Å

Label	Centroid distance	Symmetry		Coulombic energy	Polarization energy	Dispersion energy	Repulsion energy	Total energy	Contacts
A	4.501	*x* + ½, −*y* + ½, −*z*	PIXEL	−3.5	−2.3	−15.1	12.9	−7.9	Long C3H3⋯π = 3.03 Å and long C4H4⋯π 3.12 Å
D		*x* − ½, −*y* + ½, −*z*	SAPT2+3	−6.4	−2.6	−20.3	21.1	−8.2	
									
B	4.696	−*x*, *y* − ½, −*z* + ½	PIXEL	−5.0	−2.8	−15.7	13.7	−9.7	C5H5⋯π = 2.72 Å (∠ = 136°)
C		−*x*, *y* + ½, −*z* + ½	SAPT2+3	−7.2	−2.6	−19.9	19.9	−9.8	
									
E	5.949	*x* − ½, −*y* + 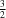 , −*z*	PIXEL	−7.9	−3.5	−12.2	12.5	−11.1	C2H2⋯N1 = 2.58 Å (∠ = 135°)
H		*x* + ½, −*y* + 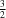 , −*z*	SAPT2+3	−10.8	−3.5	−14.6	18.1	−10.8	
									
F	5.880	−*x* + 1, *y* + ½, −*z* + ½	PIXEL	−7.1	−2.8	−10.7	8.5	−12.2	C6H6⋯N1 = 2.76 Å (∠ = 124°)
G		−*x* + 1, *y* − ½, −*z* + ½	SAPT2+3	−10.0	−2.7	−13.5	13.8	−12.4	
									
I	6.806	*x*, *y* − 1, *z*	PIXEL	0.0	−0.1	−1.1	0.0	−1.2	Long-range dispersion, H2⋯H5 = 4.75 Å
L		*x*, *y* + 1, *z*	SAPT2+3	0.0	−0.1	−1.7	0.1	−1.8	
									
J	5.392	*x* + 1, *y*, *z*	PIXEL	−1.2	−1.3	−9.0	5.4	−6.2	Highly offset stack. No overlap.
K		*x* − 1, *y*, *z*	SAPT2+3	−3.3	−1.7	−12.6	11.0	−6.6	Shortest contact, H4⋯H6 = 2.74 Å
									
M	5.856	−*x* + ½, −*y* + 1, *z* + ½	PIXEL	−0.6	−0.9	−8.6	4.7	−5.4	Non-specific dispersion, H2⋯H5 2.51 Å,
N		−*x* + ½, −*y* + 1, *z* − ½	SAPT2+3	−1.0	−0.9	−9.6	6.9	−4.6	H6⋯H2 2.69 Å and H6⋯H3 2.62 Å

**Table 4:** Comparison of the Raman lattice modes in pyridine II and pyridine III The values given for the Heyns & Venter study are taken from their Table 3 with values from their Fig. 1 in parentheses.

Pressure (GPa)	Reference	Phase assignment	Proposed phase	Lattice modes (cm^−1^)
1.0	(Heyns & Venter, 1985 ▸)	I	II	60(-), 72 (71), 85 (89), 91(-), 111 (110), 153 (152)
1.09	This study, experimental	II	II	55, 70, 89, 111, 152
1.09	This study, DFT	II	II	56, 76, 93, 116, 136 (weak), 156
1.69	This study, experimental	III	III	123, 141 (sh), 154
1.69	This study, DFT	III	III	33, 110, 148, 164
1.7	(Fanetti *et al.*, 2011 ▸)	II	III	126, 163
2.0	(Zhuravlev *et al.*, 2010 ▸)	II	III	130, 165
2.5	(Heyns & Venter, 1985 ▸)	II	II/III mixture	57 (57), 69 (69), 87 (88), 96(-), 108 (108), 142 (142), 187 (187)

**Table 5:** Displacive (Å) and rotational (radians) symmetry-modes and amplitudes for the phase III→II transition Divide by 2 to obtain corresponding molecular translation distances and rotation angles (see Section 2.9). The first mode vector corresponds to the molecule with the centroid near [0.37, 0.62, 0.625] in the unstrained child cell.

Mode name	Γ_1_(*A*_1_)	Γ_2_(*B*_2_)	Γ_2_(*B*_2_)
Norm factor	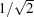	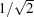	1
			
Molecular centroid	Mode vectors		
(*x*, *y*, *z*)	1 1 0	1 1 0	0 0 1
(*x* + ½, −*y* + 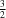 , −*z* + 1)	1 1 0	1 1 0	0 0 1
(−*x* + 1, *y* − ½, −*z* + 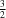 )	1 1 0	1 1 0	0 0 1
(−*x* + ½, −*y* + 1, *z* + ½)	1 1 0	1 1 0	0 0 1
			
Mode type	Mode amplitudes		
Displacive	−0.765	0.140	−0.045
Rotational	0.550	0.239	−0.342
