# Supplementary figures and images for: High-pressure polymorphism in pyridine
Source: IUCrJ. 2020 Jan 1;7(Pt 1):58–70. doi: 10.1107/S2052252519015616 (PMC6949594; doi:10.1107/S2052252519015616)

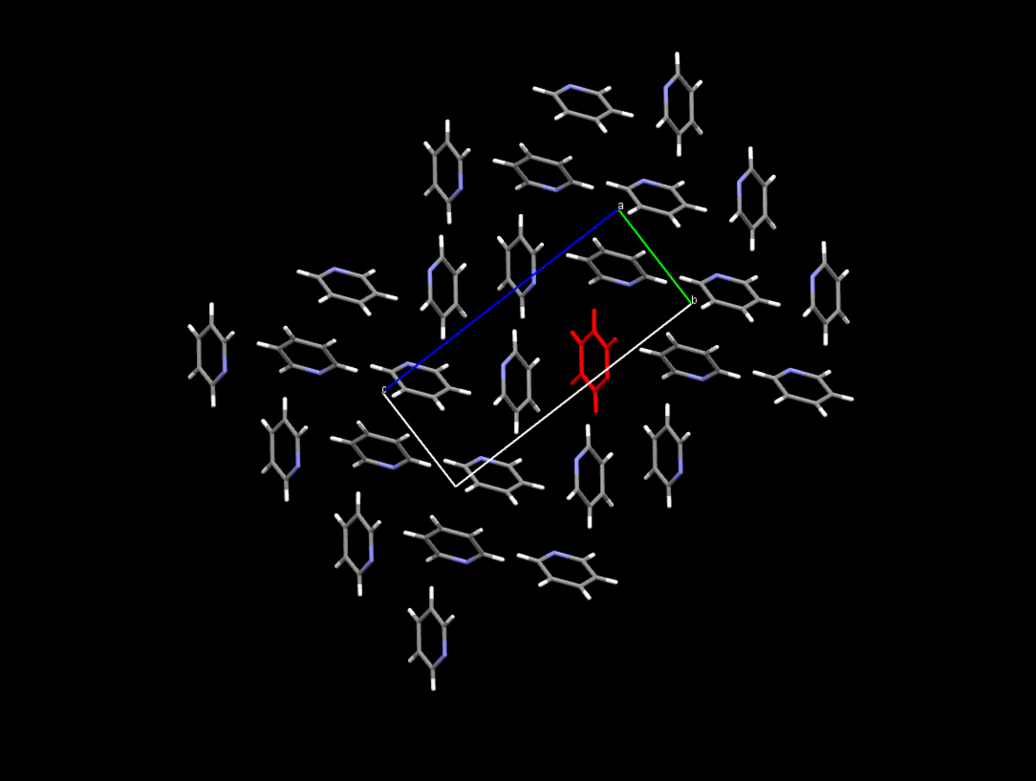

Supplement: Supplementary file 2 [file m-07-00058-sup2.gif]

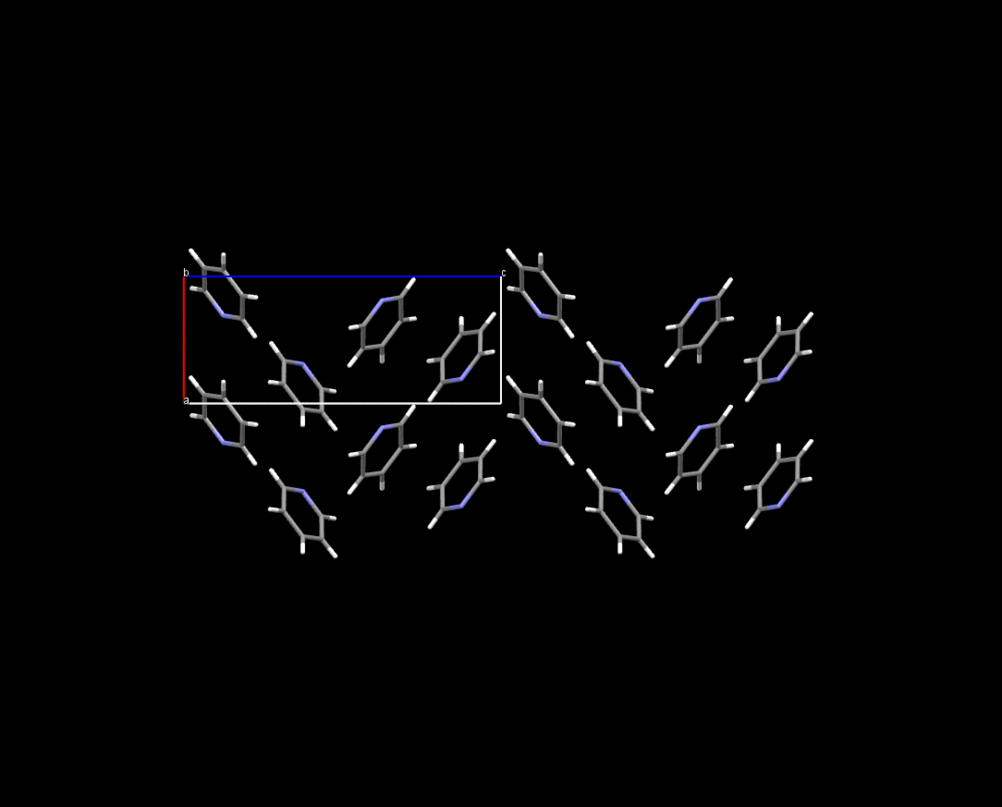

Supplement: Supplementary file 3 [file m-07-00058-sup3.gif]

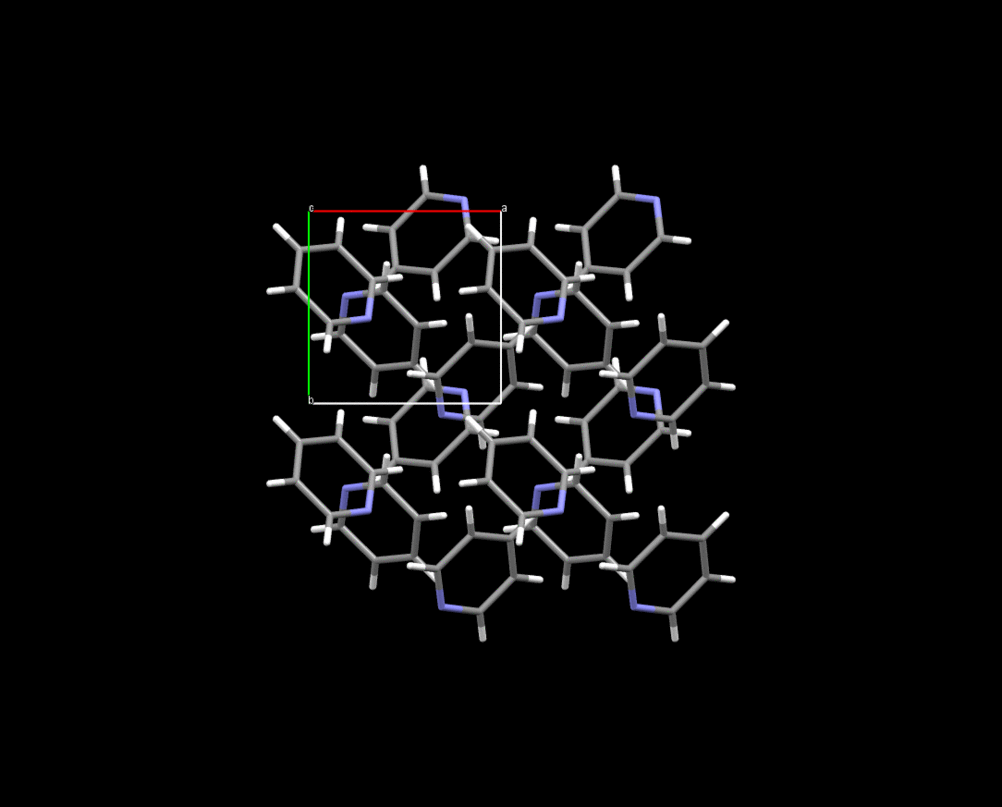

Supplement: Supplementary file 4 [file m-07-00058-sup4.gif]
