# Supplementary material for: High-pressure polymorphism in pyridine
Source: IUCrJ. 2020 Jan 1;7(Pt 1):58–70. doi: 10.1107/S2052252519015616 (PMC6949594; doi:10.1107/S2052252519015616)
Supplement: Supplementary file 5 [file m-07-00058-sup5.pdf]

IUCrJ

Volume 7 (2020)

Supporting information for article:

High-pressure polymorphism in pyridine

Nico Giordano, Eugene Gregoryanz, Miriam Peña Alvarez, Cara E. Vennari, Willliam G. Marshall, Václav Eigner, Christine M. Beavers, Simon J. Teat, Nicholas P. Funnell and Simon Parsons

Contents

Figure S1. The full Raman spectrum of domain 2 in the phase-II sample from which diffraction data were collected.

Figure S2. The full Raman spectrum of domain 1 in the phase-III sample from which diffraction data were collected.

Figure S3. Raman spectra collected from five domains in phase-II.

Figure S4. Raman spectra collected from five domains in phase-III.

Figure S5. Contacts A-N in the first coordination sphere of (a) pyridine-III and (b) pyridine-II. Short intermolecular interactions are shown as blue dashed lines.

Movies. A separate files are available containing animations (animated gifs) of the pyridine III to II phase transition viewed along the crystallographic *a*, *b* and *c* axes. The red molecule in the *a*-axis plot is the central molecule of the coordination sphere plots of Fig. 2 in the main text. The amplitudes derived from the symmetry mode analysis vary linearly over the course of the transition. The linearity is an assumption made for the purpose of generating the animation, and should not be interpreted as showing the 'mechanism' for the transition.

Crystallographic Information Files. Cifs containing the final structural models and the single-crystal X-ray intensity data for phases II and III are available in a separate file.

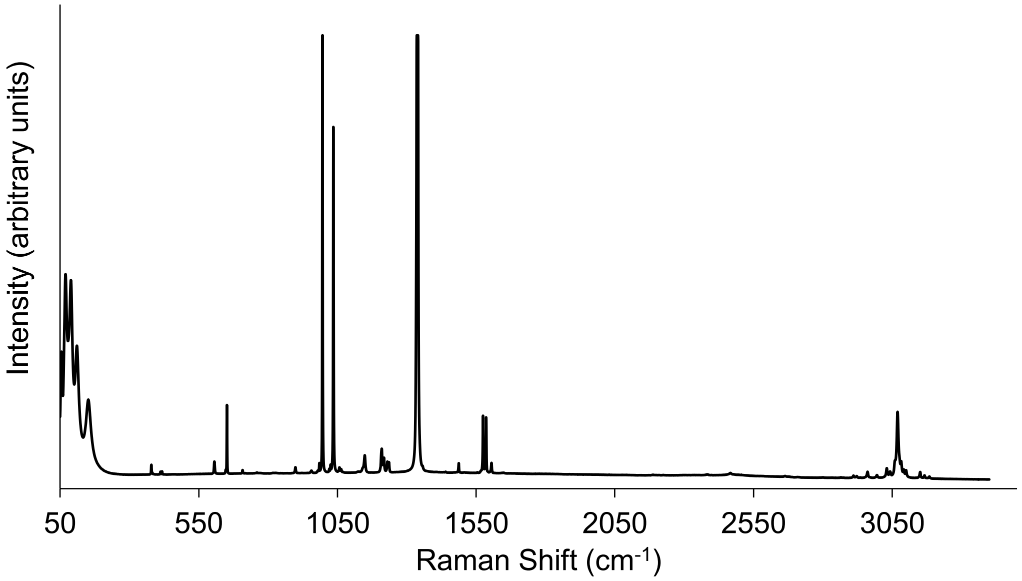

Figure S1 The full Raman spectrum of domain 2 in the phase-II sample from which diffraction data were collected.

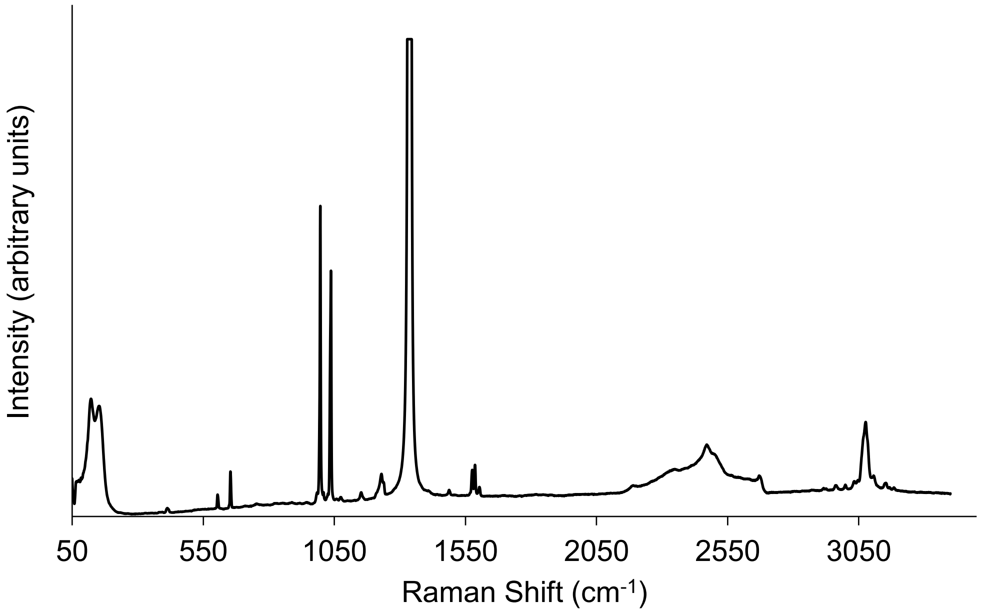

Figure S2 The full Raman spectrum of domain 1 in the phase-III sample from which diffraction data were collected.

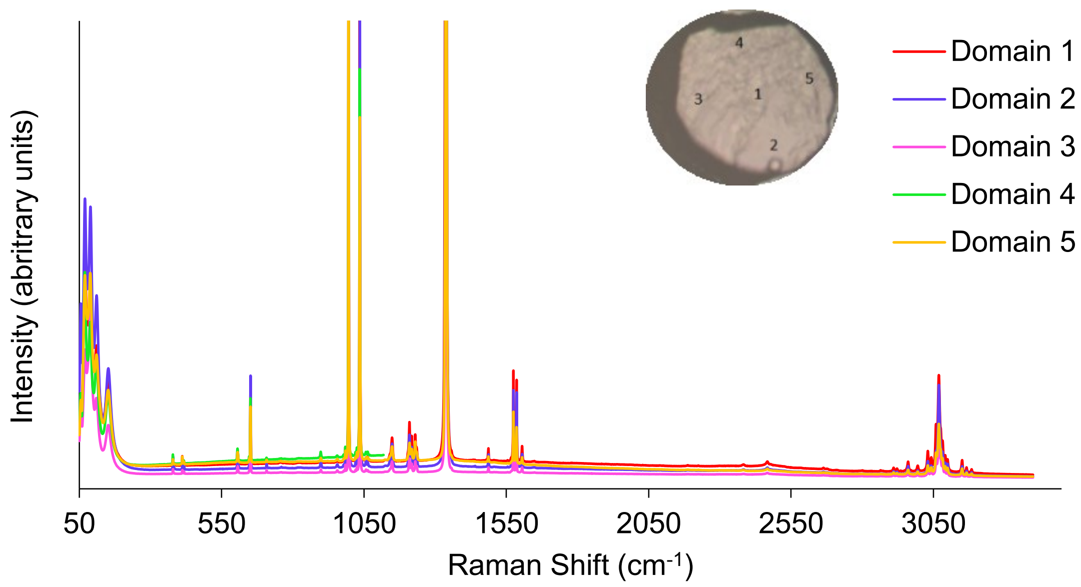

Figure S3 Raman spectra collected from five domains in phase-II.

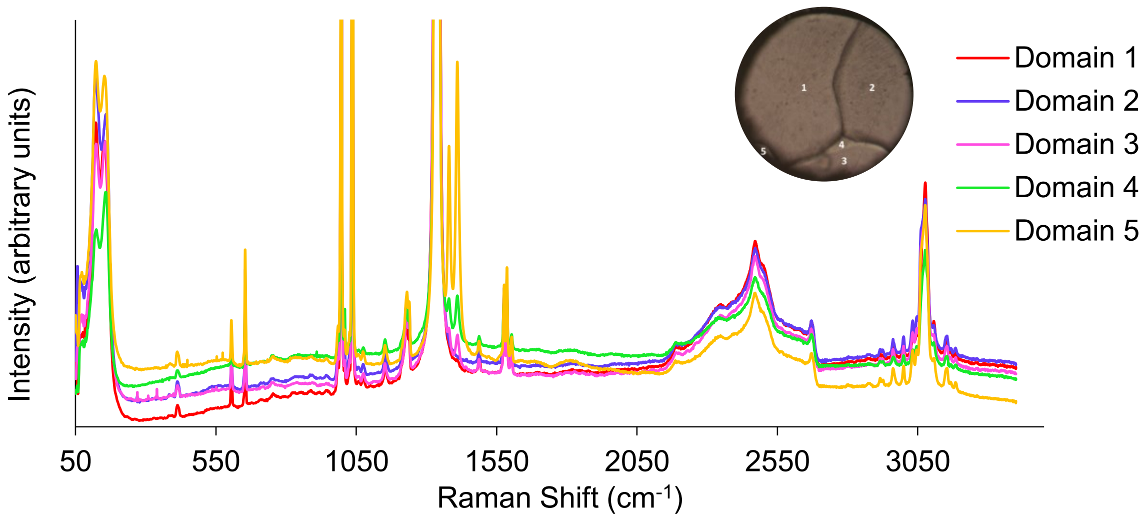

Figure S4 Raman spectra collected from five domains in phase-III.

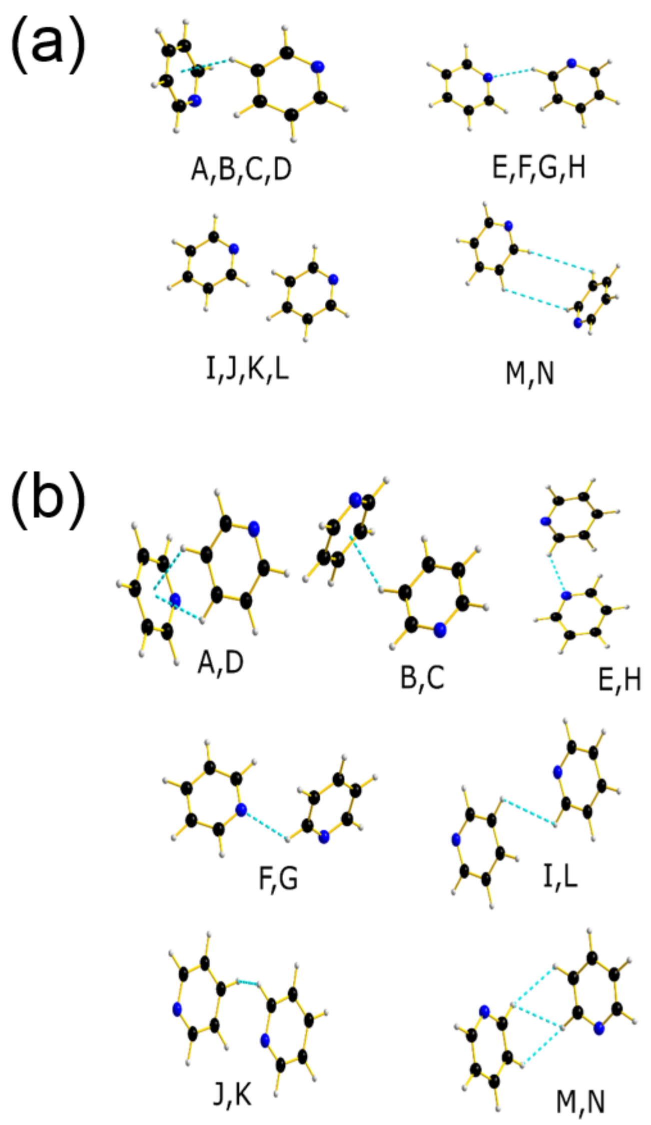

Figure S5 Contacts A-N in the first coordination sphere of (a) pyridine-III and (b) pyridine-II. Short intermolecular interactions are shown as blue dashed lines.
